# Protocol for identifying DNA damage responders through proximity biotinylation of proliferating cell nuclear antigen interactors

**DOI:** 10.1016/j.xpro.2025.104136

**Published:** 2025-10-10

**Authors:** Roberta Borosta, István Szepesi-Nagy, István Kató, Gergely Róna

**Affiliations:** 1 MTA-HUN-REN RCNS Lendulet “Momentum” DNA Repair Research Group, Institute of Molecular Life Sciences, HUN-REN Research Centre for Natural Sciences, 1117 Budapest, Hungary; 2 Doctoral School of Biology, Faculty of Science, Eötvös Loránd University, 1117 Budapest, Hungary; 3 Doctoral School of Semmelweis University, 1085 Budapest, Hungary; 4 Obuda University, Doctoral School of Applied Informatics and Applied Mathematics, 1034 Budapest, Hungary; 5 Department of Biochemistry and Molecular Pharmacology at NYU Grossman School of Medicine, New York NY 10016, USA

**Keywords:** Cell Biology, Cell separation/fractionation, Signal transduction

## Abstract

TurboID-based proximity labeling is a powerful approach to capture protein-protein interactions within their native cellular environment. Here, we present a step-by-step protocol for fusing proliferating cell nuclear antigen (PCNA) to TurboID and generating stable cell lines via lentiviral transduction. We describe steps for cell synchronization, DNA damage induction, and proximity labeling, followed by fractionation, affinity purification, and mass spectrometry to identify biotinylated proteins.

For complete details on the use and execution of this protocol, please refer to Rona et al.^1^

## Highlights

• A TurboID-based proximity labeling protocol to identify proteins involved in DNA repair
• Instructions for biochemical fractionation to reduce background and sample complexity
• Steps to efficiently synchronize cells into different cell cycle phases
• Streptavidin pull-down isolates biotinylated interactors for MS

## Abstract (editor highlights)

Publisher’s note: Undertaking any experimental protocol requires adherence to local institutional guidelines for laboratory safety and ethics.

## Graphical abstract

**Figure:**
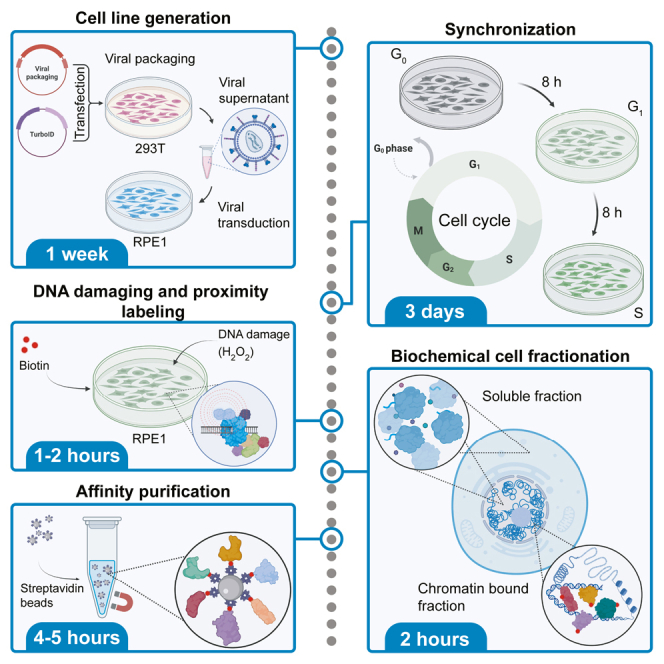

## Before you begin

The following protocol outlines a step-by-step procedure for applying proximity labeling to investigate the interactome of chromatin bound proliferating cell nuclear antigen (PCNA) in living cells. In our study, we employed this approach in G1-synchronized RPE1 cells to characterize PCNA-associated proteins in response to oxidative DNA damage.^1^ However, the protocol is highly versatile and can be readily adapted to other cell types, proximity labeling targets, and genotoxic stress conditions. It is also compatible with asynchronous cell populations or cells synchronized at other phases of the cell cycle, enabling broader exploration of dynamic protein interactions across various biological contexts.

### Innovation

While PCNA has long been recognized as a central hub in coordinating replication and repair processes, traditional methods, such as co-immunoprecipitation, often capture only stable interactions and may disrupt protein complexes. In contrast, TurboID enables efficient, proximity-dependent biotinylation of proteins in living cells, preserving transient and context-specific interactions. This approach is particularly powerful for investigating how PCNA-associated networks change under stress, for example during oxidative DNA damage induced by H_2_O_2_. By allowing time-resolved and unbiased labeling, the protocol can reveal regulatory dynamics that cannot be detected by conventional assays.

### Obtain necessary plasmids

**Timing: 1 week**

All plasmids used in this protocol, including those referenced in the original publication,^1^ on which this procedure is based, have been deposited to Addgene. Before beginning the protocol, please ensure you have obtained the necessary plasmids from Addgene (https://www.addgene.org/browse/article/28244043/, https://www.addgene.org/viral-vectors/).

For the expression of NLS-HA-TurboID-PCNA, we recommend using a lentiviral vector, driven by a strong promoter, such as CMV, to ensure adequate expression, similar to endogenous PCNA levels. To match these expression levels with the control NLS-HA-TurboID construct, we suggest using a vector with a weaker promoter, such as the retroviral pBABE vector.

1. For the generation of stable cell lines expressing NLS-HA-TurboID-PCNA, and the control NLS-HA-TurboID, the following plasmids will need to be obtained from Addgene:
  a. psPAX2 (Addgene #12260) for lentiviral packaging; psPAX2 was a gift from Didier Trono (School of Life Sciences, Ecole Polytechnique Fédérale de Lausanne (EPFL), 1015, Lausanne, Switzerland).
  b. pCMV-VSV-G (Addgene #8454) for lentiviral or retroviral packaging; pCMV-VSV-G was a gift from Bob Weinberg^2^ (Whitehead Institute for Biomedical Research, Cambridge, MA 02142, USA; Department of Biology, Massachusetts Institute of Technology, Cambridge, MA 02142, USA; MIT Ludwig Center for Molecular Oncology, Cambridge, MA 02139, USA).
  c. pUMVC (Addgene #8449) for retroviral packaging; pUMVC was a gift from Bob Weinberg.^2^
  d. pBABE-NLS-HA-TurboID (Addgene #215073) to express NLS-HA-TurboID form a retroviral vector; pBABE-NLS-HA-TurboID was a gift from Michele Pagano^1^ (Department of Biochemistry and Molecular Pharmacology, NYU Grossman School of Medicine, New York, NY 10016, USA; Laura and Isaac Perlmutter Cancer Center, NYU Grossman School of Medicine, New York, NY 10016, USA; Howard Hughes Medical Institute, NYU Grossman School of Medicine, New York, NY 10016, USA).
  e. pLVX-NLS-HA-TurboID-PCNA (Addgene #215076) to express NLS-HA-TurboID-PCNA from a lentiviral vector; pLVX-NLS-HA-TurboID-PCNA was a gift from Michele Pagano.^1^
2. Amplify, then purify the plasmids above using any commercially available endotoxin-free plasmid DNA isolation kit following the manufacturer’s guidelines. In this protocol we have used the ZymoPURE Plasmid Miniprep Kit (Zymo Research).

### Generation of stable RPE1 cell lines

**Timing: 1 week**

For BSL-2 safety measures while working with recombinant viruses, please refer to: “NIH Guidelines, Section III -D-3: Recombinant viruses in tissue culture” (https://osp.od.nih.gov/wp-content/uploads/NIH_Guidelines.pdf). Prior to experimentation, routinely test cells for mycoplasma contamination with any commercially available mycoplasma detection kit following the manufacturer’s recommendation. In this protocol we have used the MyStrip Mycoplasma Detection Kit (InvivoGen).

3. On the first day seed ∼4 × 10^6^ HEK293T into a 10 cm culture dish in 10 mL culture media (Complete DMEM). Prepare two 10 cm culture dishes like this, to have a separate plate for both the control NLS-HA-TurboID and the NLS-HA-TurboID-PCNA constructs.

***Note:*** This cell number enables a ∼65%–80% confluency by the following day.

4. On the second day, each of the 10 cm culture dish prepared in Step 3 will be either transfected with the NLS-HA-TurboID encoding retroviral vector or the NLS-HA-TurboID-PCNA encoding lentiviral vector. For each of the constructs, add 250-250 μL of Opti-MEM reduced serum medium into two separate sterile 1.5 mL microcentrifuge tubes.
5. To pack the NLS-HA-TurboID-PCNA lentiviral vector, combine 1.5 μg of psPAX2 (Addgene #12260), 1.5 μg of pCMV-VSV-G (Addgene #8454) vectors along with 3 μg of the pLVX-NLS-HA-TurboID-PCNA (Addgene #215076) vector in one of the 1.5 mL microcentrifuge tubes prepared in Step 4.
6. To pack the NLS-HA-TurboID retroviral vector, combine 1.5 μg of pUMVC (Addgene #8449), 1.5 μg of pCMV-VSV-G (Addgene #8454) vectors along with 3 μg of pBABE-NLS-HA-TurboID (Addgene #215073) vector in one of the 1.5 mL microcentrifuge tubes prepared in Step 4.

***Note:*** From this point onward, the protocol proceeds identically for both types of viral vectors (retro- and lentiviral).

7. Add 1 μL of P3000 reagent (Lipofectamine 3000 Transfection Reagent, Thermo Fisher Scientific) for each μg of DNA added into the Opti-MEM/DNA mixture (in this case 6-6 μL for the two tubes from Step 5 and 6) and mix gently by tapping.

***Note:*** Do not vortex or pipette up and down to limit the mechanical shedding of the plasmid DNA.

8. In another 1.5 mL microcentrifuge tube, combine 2 μL Lipofectamine 3000 (Lipofectamine 3000 Transfection Reagent, Thermo Fisher Scientific) per μg DNA (in this case, 12-12 μL for the two tubes from Step 5 and 6) with 500 μL of Opti-MEM reduced serum media.

***Note:*** This mixture will be used for both the NLS-HA-TurboID-PCNA and the NLS-HA-TurboID constructs.

9. Add 250-250 μL of the Lipofectamine 3000-Opti-MEM mixture from Step 8 to each tube containing the plasmid DNA prepared in Step 5 and 6. Incubate the mixtures at room temperature (RT, is defined as 22°C–25°C throughout the protocol) for 15 minutes to allow complex formation.

***Note:*** Mix the plasmid DNA (Step 7) with the transfection reagent in Opti-MEM (Step 8) only by gentle tapping with your index finger and not by vortexing. Harsh mechanical mixing will break the DNA-liposome complexes.

10. Carefully add the two different transfection mixtures dropwise to the two plates of seeded HEK293T cells prepared in Step 3.

**CRITICAL:** Ensure that the cells are not disturbed or detached during the process. Gently swirl the plates to evenly distribute the complexes.

11. Let the HEK293T cells produce the desired retro- and lentiviral particles for 72 hours in a cell culture incubator, at 37°C and 5% CO_2_.
12. Collect the virus-containing supernatant from HEK293T cells 72 hours post-transfection into a 50 mL centrifuge tube.
13. Filter the supernatant (∼10 mL) through a 0.45 μm sterile Millex filter unit into a fresh 50 mL centrifuge tube to remove cell debris and any detached cells.

***Optional:*** To enhance viral infection efficiency, add polybrene (hexadimethrine bromide), to a final concentration: 8 μg/mL. Prepare an 8 mg/mL polybrene stock solution in ddH_2_O, filter sterilize it through a 0.2 μm Millex filter unit.

***Note:*** The prepared virus containing supernatant can be stored up to a week at 4°C when protected from light (by wrapping it in foil, or by placing it in a non-transparent storage box).

14. To infect RPE1 cells, seed ∼3 × 10^6^ RPE1 cells into a 10 cm dish at least 16 hours prior to infection.
15. 16–24 hours after plating the cells, carefully add the viral supernatant (prepared in Step 13) to the RPE1 cells prepared in Step 14.
16. Incubate the cells with the viral supernatant for 6–16 hours.
  a. Following infection, aspirate and discard the viral medium.
  b. Add 10 mL fresh growth medium/plate (Complete DMEM).

***Note:*** To fine-tune transgene expression levels, infect cells using a series of viral dilutions while maintaining a constant incubation time, ideally between 6–16 h. For consistency, use the same incubation time across all dilutions. After establishing stable cell lines, assess transgene expression by western blot and compare it to the endogenous protein levels.

17. 48 hours post-infection start puromycin selection at a final concentration of 1–2 μg/mL for 2–4 days, or until non-infected control cells are eliminated (can take up to 4 days).

### Verification of expression of TurboID-tagged PCNA and controls

**Timing: 2 days**

Before proceeding with the downstream protocol, it is essential to confirm that the stable cell lines generated after puromycin selection express the desired constructs at comparable levels. Expression of NLS-HA-TurboID and NLS-HA-TurboID-PCNA can be assessed using an anti-HA antibody. The expected molecular weight of NLS-HA-TurboID is approximately 40 kDa, while NLS-HA-TurboID-PCNA is expected to be around 73.8 kDa. The procedure for evaluating target gene expression by Western blot is outlined below.

18. Collect around 0.5 x 10^6^ RPE1 cells of each stable cell line to lyse with trypsinization or cell scraping. Centrifuge the cells with 300 g x 5 min at 4°C.
19. Wash the pellet twice with 500 μL DPBS to remove FBS components. Discard the supernatant.
20. Resuspend the pellet in 300 μL RIPA IP buffer to lyse cells by incubating on ice for 20 min.
21. Remove insoluble fraction by centrifuging the sample at 20,000 g x 10 min at 4°C.
  a. Carefully transfer the supernatant into a fresh 1.5 mL microcentrifuge tube by using a P1000 pipette, ensuring that the pellet remains undisturbed.
  b. Discard the pellet.
22. Measure and normalize the protein concentration of each sample with BCA method, following the manufacturers’ recommendation.
23. For subsequent steps, please follow the Western blotting protocol established by your laboratory using an HA-antibody to detect the expression of the genes of interest.

***Note:*** Load between 10 and 40 μg of lysate/lane. Expect NLS-HA-TurboID to run at 40 kDa, while NLS-HA-TurboID-PCNA is expected to run at 73.8 kDa.

## Key resources table

**Table:** 

REAGENT or RESOURCE	SOURCE	IDENTIFIER
**Antibodies**		

Rabbit anti-HA Tag antibody (WB and IF, 1:2,000)	Bethyl Laboratories	A190-108A; RRID: AB_67465

**Chemicals, peptides, and recombinant proteins**		

2-mercaptoethanol	Sigma-Aldrich	M3148-25ML
Biotin	Sigma-Aldrich	B4501-100MG
Complete ULTRA Tablets	Roche	5892953001
D-Sucrose	Thermo Fisher Scientific	bp220-1
DMEM, high glucose	Thermo Fisher Scientific	11965092
DPBS, no calcium, no magnesium	Thermo Fisher Scientific	14190144
DTT	VWR	441494N
Dynabeads MyOne C1 Streptavidin Magnetic Beads	Thermo Fisher Scientific	65001
EDTA, (0.5 M, pH 8.0)	Thermo Fisher Scientific	15575020
EGTA, (0.5 M, pH 8.0)	Thermo Fisher Scientific	J61721.AD
FBS	Sigma-Aldrich	F9665-500ML
FBS, dialyzed	Thermo Fisher Scientific	26400044
Hydrogen peroxide solution	Sigma-Aldrich	H1009-100ML
Magnesium chloride solution (1 M)	Sigma-Aldrich	M1028-100ML
N-Ethylmaleimide	Thermo Fisher Scientific	L00355.09
NP-40 Surfact-Amps	Thermo Fisher Scientific	28324
NuPAGE LDS Sample Buffer (4X)	Thermo Fisher Scientific	NP0008
Penicillin/streptomycin solution	Corning Life Sciences	30-001-CI
PhosSTOP	Roche	4906845001
PIPES buffer (0.5 M, pH 7.0)	Bioworld	41620034–1
PMSF	Roche	10837091001
Polybrene (hexadimethrine bromide)	Sigma-Aldrich	TR-1003
Puromycin (10 mg/mL)	Sigma-Aldrich	P9620
Reduced serum media (Opti-MEM)	Thermo Fisher Scientific	31985070
SDS (10%)	Thermo Fisher Scientific	15553027
Sodium chloride solution (5 M)	Sigma-Aldrich	S5150
Sodium deoxycholate	Sigma-Aldrich	D6750-10G
TRIS-HCl (1 M, pH 7.5)	Thermo Fisher Scientific	15567027
Triton X-100 aqueous solution (10%)	Sigma-Aldrich	11332481001
Trypsin-EDTA (0.5%), no phenol red	Thermo Fisher Scientific	1540054
TurboNuclease	Accelagen	NC0298896

**Critical commercial assays**		

Lipofectamine 3000 Transfection Reagent	Thermo Fisher Scientific	L3000008
MyStrip Mycoplasma Detection Kit	InvivoGen	rep-mys-10
Pierce BCA Protein Assay Kit	Thermo Fisher Scientific	A65453
ZymoPURE Plasmid Miniprep Kit	Zymo Research	D4209

**Deposited data**		

Mendeley Data: Raw, uncropped supporting western blot files for: Borosta R. Rona G. “Protocol for identifying DNA damage responders through proximity biotinylation of proliferating cell nuclear antigen interactors”	Mendeley Data	Mendeley Data, V1, https://doi.org/10.17632/4gxswncytm.1

**Experimental models: Cell lines**		

Human: HEK 293T (female)	ATCC	CRL-3216 RRID:CVCL_0063
Human: hTERT RPE1 (female)	ATCC	CRL-4000, RRID:CVCL_4388

**Recombinant DNA**		

pBABE-NLS-HA-TurboID	Addgene	Addgene #215073, RRID:Addgene_215073
pCMV-VSV-G	Addgene	Addgene #8454, RRID:Addgene_8454
pLVX-NLS-HA-TurboID-PCNA	Addgene	Addgene #215076, RRID:Addgene_215076
psPAX2	Addgene	Addgene #12260, RRID:Addgene_12260
pUMVC	Addgene	Addgene #8449, RRID:Addgene_8449

**Other**		

DynaMag-2 Magnet magnetic stand	Thermo Fisher Scientific	12321D
Millex-filter unit pore size 0.45 μm	MilliporeSigma	SLHVR33RS
Millex-filter unit pore size 0.22 μm	MilliporeSigma	SLGVR33RS

## Materials and equipment

**Table:** CSK lysis buffer (pH 7.0)

Reagent	Final concentration	Stock solution	Add to 10 mL
PIPES	10 mM	0.5 M (pH 7.0)	200 μL
Triton X-100	0.1%	10%	100 μL
NaCl	100 mM	5 M	200 μL
Sucrose	300 mM	342.30 g/mol	1.027 g
MgCl_2_	3 mM	1 M	30 μL
EGTA	1 mM	0.5 M (pH 8.0)	20 μL
DTT	1 mM	1 M	10 μL
N-Ethylmaleimide	5 mM	125.13 g/mol	6.257 mg
Complete ULTRA Tablets	1 tablet / 10 mL	1 tablet / 10 mL	1 tablet
PhosSTOP	1 tablet / 10 mL	1 tablet / 10 mL	1 tablet
ddH_2_O	N/A	N/A	adjust volume to 10 mL

Store at 4°C for up to 6 months. Always add DTT, N-Ethylmaleimide, Complete ULTRA Tablets and PhosSTOP freshly to the solution, just before use.

**Table:** RIPA IP buffer (pH 7.5)

Reagent	Final concentration	Stock solution	Add to 10 mL
TRIS-HCl	25 mM	1 M (pH 7.5)	250 μL
NaCl	250 mM	5 M	500 μL
NP-40	1%	10%	1 mL
Sodium deoxycholate	1%	414.55 g/mol	100 mg
SDS	0.1%	10%	100 μL
EGTA	1 mM	0.5 M (pH 8.0)	20 μL
DTT	1 mM	1 M	10 μL
N-Ethylmaleimide	5 mM	125.13 g/mol	6.257 mg
Complete ULTRA Tablets	1 tablet / 10 mL	1 tablet / 10 mL	1 tablet
PhosSTOP	1 tablet / 10 mL	1 tablet / 10 mL	1 tablet
ddH_2_O	N/A	N/A	adjust volume to 10 mL

Store at 4°C for up to 6 months. Always add DTT, N-Ethylmaleimide, Complete ULTRA Tablets and PhosSTOP freshly to the solution, just before use.

**Table:** High-salt RIPA IP buffer

Reagent	Final concentration	Stock solution	Add to 10 mL
TRIS-HCl	25 mM	1 M (pH 7.5)	250 μL
NaCl	500 mM	5 M	1 mL
NP-40	1%	10%	1 mL
Sodium deoxycholate	1%	414.55 g/mol	100 mg
SDS	0.1%	10%	100 μL
EGTA	1 mM	0.5 M (pH 8.0)	20 μL
DTT	1 mM	1 M	10 μL
N-Ethylmaleimide	5 mM	125.13 g/mol	6.257 mg
Complete ULTRA Tablets	1 tablet / 10 mL	1 tablet / 10 mL	1 tablet
PhosSTOP	1 tablet / 10 mL	1 tablet / 10 mL	1 tablet
ddH_2_O	N/A	N/A	adjust volume to 10 mL

Store at 4°C for up to 6 months. Always add DTT, N-Ethylmaleimide, Complete ULTRA Tablets and PhosSTOP freshly to the solution, just before use.

**Table:** Elution buffer

Reagent	Final concentration	Stock solution	Add to 10 mL
TRIS-HCl	70 mM	1 M (pH 7.5)	700 μL
SDS	2%	10%	2 mL
EDTA	0.5 mM	0.5 M (pH 8.0)	10 μL
2-mercaptoethanol	350 mM	14230 mM	245.96 μL
ddH_2_O	N/A	N/A	adjust volume to 10 mL

Prepare fresh, just before use. Keep it at room temperature to avoid any precipitation of the SDS. For multiple samples, adjust the reagent volumes accordingly.

**Table:** Other solutions

Name	Reagents
Complete DMEM	DMEM, 10% FBS, 1% penicillin/streptomycin
Complete biotin-free DMEM	DMEM, 10% dialyzed FBS, 1% penicillin/streptomycin
Serum and biotin-free DMEM	DMEM, 1% penicillin/streptomycin

If your cell lines are sensitive, add 0.01% dialyzed FBS optionally to the serum and biotin-free DMEM to ensure their survival. Store the media at 4°C for up to 3 months and bring it to 37°C before adding it to the cells.

**CRITICAL:** As SDS is an irritant to the skin, eyes, and respiratory tract, always wear gloves, a lab coat, and protective eyewear when handling it. If you are working with SDS powder, use a fume hood to avoid inhalation.

**CRITICAL:** As DTT and 2-mercaptoethanol are harmful if inhaled or absorbed through the skin and may cause irritation, always wear gloves, a lab coat, and protective eyewear when handling it. If you are working with DTT powder, use a fume hood to avoid inhalation. As DTT is sensitive to air and light, prepare it fresh when possible, and store stock solutions in tightly sealed aliquots, wrapped in foil at −20°C.

## Step-by-step method details

### Synchronizing RPE1 cells into G1

**Timing: 3 days**

To specifically investigate PCNA’s role in DNA repair during the G1 phase, distinct from its well-known function in replication during S phase, we used the following protocol to synchronize RPE1 cells in G1. The protocol describes how to generate one 15 cm dish of G1-synchronized RPE1 cells. Be sure to prepare enough plates to cover all experimental conditions, including treatments and replicates. When planning, we recommend comparing the expression of your protein of interest (POI) to endogenous levels by Western blot. Depending on the POI expression level, multiple 15 cm dishes may be required per sample. As a general guideline, use 2-4 plates per condition when POI expression is similar to endogenous, up to 8 plates when expression is much lower, and 1-2 plates when it is much higher. This will vary between proteins and should be optimized by the user. For PCNA, we obtained the best results when viral expression levels were close to endogenous, which helped minimize potential overexpression artifacts.

***Optional:*** Maintain cells in complete biotin-free DMEM for 4 days prior to initiating serum starvation. This step reduces the amount of endogenously biotinylated proteins which will result in less background. This is particularly useful if the biotin induced proximity labeling is only allowed for a limited time (few hours) or if the expression of the TurboID-tagged proteins is weak.

1. Prepare RPE1 cells for serum starvation.
  a. Wash the RPE1 cells once with 5 mL DPBS.
  b. Remove the DPBS by pipetting or vacuum aspiration.
2. Detach RPE1 cells using 2 mL of 0.05% trypsin / plate.

***Note:*** A fully confluent 15 cm dish will have a yield of around 6–7.5 × 10^6^ RPE1 cells after these washing steps. Start with enough plates to have an adequate number of cells for all your samples. You will need to be able to plate ∼5–6 × 10^6^ cells at Step 5.

3. Inactivate the trypsin by transferring the detached cells into 10 mL of complete biotin-free DMEM in 15 mL centrifuge tubes.
4. Centrifuge the cells at 150 × g, RT for 5 min.
  a. Remove the supernatant and wash the cells with 10 mL of DPBS.
  b. Repeat this washing step with DPBS twice.
5. After the final wash, resuspend the cells in 10 mL of serum and biotin-free DMEM.
  a. Count them on a cell counter.
  b. Plate ∼5–6 × 10^6^ cells onto a 15 cm tissue culture dish.

***Note:*** After careful visual observation, the cell confluency should be around 40% once the cells attach.

***Note:*** If the cells tend to clump, resuspend them in 1 mL of serum and biotin-free DMEM and pipette up and down five times using a P1000 tip to break up the clumps, then dilute the suspension to a final volume of 10 mL.

6. Culture the cells for 72 hours. The cells will then be in G0 phase.

***Note:*** Cells should gradually stop proliferating after 24 h. By careful visual observation the confluency of the plate after 72 h should not exceed 90%.

***Note:*** If your experiment requires cells to be in G0 you can directly proceed with the “Induction of DNA damage in cells and proximity labeling” step.

7. Release the synchronized cells into G1.
  a. Detach the serum-starved RPE1 cells from the 15 cm plates using 2 mL of 0.05% trypsin.
  b. Wash the cells with DPBS for 2 minutes at 37°C.

***Note:*** Cells detach more rapidly after serum starvation. Use caution during the DPBS wash and limit trypsinization time to avoid cell damage.

8. Inactivate the trypsin by transferring the cells into 10 mL of complete biotin-free DMEM in 15 mL centrifuge tubes.
9. Centrifuge the cells at 150 g x 5 min, at RT. Remove the supernatant and resuspend all the cells in 20 mL of complete biotin-free DMEM.
10. Replate all the cells into 15 cm plates and culture them for 8-10 hours. The cells are now in G1.

***Note:*** If your experiment requires cells to be in S phase, adjust Step 10 by extending the incubation to 20 h instead of 8–10 h.

### Induction of DNA damage in cells and proximity labeling

**Timing: 1–2 h**

To detect PCNA-interacting partners in response to DNA damage, we next apply the proximity labeling assay. In this system, PCNA is expressed from a vector as a fusion protein with the biotin ligase enzyme TurboID. Using this approach, we perform biotin labeling in the presence of H_2_O_2_-induced DNA damage to selectively label PCNA-interacting proteins involved in DNA repair during G1 phase.

***Note:*** The number of plates can be scaled up according to downstream experimental requirements. The protocol outlined below describes the processing of a single 15 cm plate of G1 synchronized RPE1 cells.

11. To induce proximity biotinylation, prepare a 1 M biotin stock solution, diluted in DMSO.
  a. Add biotin at a final concentration of 50 μM to the G1 synchronized RPE1 cells prepared at Step 10.
  b. Incubate for 15 minutes.

***Note:*** To prepare a 1 M biotin stock solution, add 244,31 mg biotin to 1 mL DMSO. Aliquot and store at −20°C for up to 6 months.

12. To induce oxidative DNA damage, add 250 μM of H_2_O_2_ to the cells and incubate them for an additional 60–90 minutes.

***Note:*** H_2_O_2_ treatment is used to induce oxidative DNA lesions and activate PCNA-dependent repair pathways, such as base excision repair (BER) and mismatch repair (MMR). Adding biotin 15 min before DNA damage induction ensures that there is adequate amount of biotin already present within the cells when DNA damage is induced.

**CRITICAL:** H_2_O_2_ is a strong oxidizing agent that can cause skin and eye irritation and could be harmful if inhaled. Always handle it with care, and wear appropriate personal protective equipment including gloves, a lab coat, and eyewear.

13. Collect the RPE1 cells after treatments.
  a. Discard the media on the cells and wash cells once with 10 mL cold DPBS.
  b. Aspirate the DPBS completely by pipetting or vacuum aspiration.

***Note:*** Washing should be thorough, swirl the plate gently to ensure even washing across the entire surface, but without disturbing the cell monolayer.

**CRITICAL:** It is important to thoroughly wash off the biotin-containing medium to minimize the risk of nonspecific labeling. Additionally, removing excess biotin helps prevent interference with downstream applications.

14. Scrape cells in 3 mL DPBS that contains 1 mM freshly added PMSF and transfer them to a 15 mL conical tube.

**CRITICAL:** PMSF is a highly toxic neurotoxin and must be handled with care. Before use, remove the stock solution, diluted in ethanol, from the −20°C freezer, and warm the tube in your gloved hands, as PMSF tends to crystallize when cold and needs to be fully re-dissolved. Alternate between gently vortexing and warming the tube until the solution becomes clear. Always add PMSF just before use, as it rapidly loses activity in aqueous solutions. Mix it directly into your buffer to prevent precipitation.

15. Centrifuge the cells at 300 g x 5 min, at 4°C and remove the supernatant.

**Pause Point:** At this stage, you may either proceed with the protocol or snap-freeze the cells and store them at −80°C for up to 3 months.

### Biochemical cell fractionation into soluble and chromatin-bound fractions

**Timing: 2 h**

To specifically analyze proteins interacting with chromatin-bound PCNA during DNA repair, it is essential to fractionate cells into soluble and chromatin-associated pools. Since PCNA functions primarily on chromatin during repair, isolating this fraction enriches for relevant interactions and reduces background from soluble proteins. Nuclease treatment of the chromatin fraction further digests nucleic acids, enhancing the recovery of PCNA-associated complexes for streptavidin enrichment and mass spectrometry.

16. Lyse cells in 2 mL of CSK lysis buffer / sample for 10 minutes on ice to extract the soluble proteins.
17. Centrifuge the samples at 3,000 g x 3 min at 4°C.
18. Collect the supernatant and transfer it to a fresh tube.
  a. Keep at least 5% of this supernatant and supplement it with 4X Laemmli buffer (NuPAGE LDS Sample Buffer (4X)).
  b. Keep the pellet for downstream steps.
19. Wash the pellet by resuspending it in 1 mL of CSK lysis buffer / sample.
20. Centrifuge the samples at 3,000 g x 3 min at 4°C. Discard the supernatant and keep the pellet for downstream steps.
21. To extract chromatin bound proteins, resuspend the pellet in 1 mL of RIPA IP buffer / sample.
22. Add 3 μL (750 units) of TurboNuclease / sample and pipette up and down until samples are homogenous.
23. Incubate the samples for 30 minutes on ice with occasional gentle mixing.
24. Centrifuge the samples at 16,000 g x 15 min at 4°C.
25. Collect the supernatant and transfer it to a fresh tube.
  a. Take 5% of this supernatant and supplement it with 4X Laemmli buffer in a separate tube.
  b. The remaining lysate will be used for affinity purification.

***Note:*** The Laemmli buffer-supplemented soluble (Step 18) and chromatin-bound (Step 25) protein fractions will be used for quality control by western blot to confirm successful cellular fractionation. We recommend setting aside ∼5% of each fraction for this purpose, as this amount is sufficient to run representative quality control blots while preserving the majority of the sample for affinity purification and MS analysis.

### Affinity purification of biotinylated proteins

**Timing: 4–5 h**

**Figure 1:**
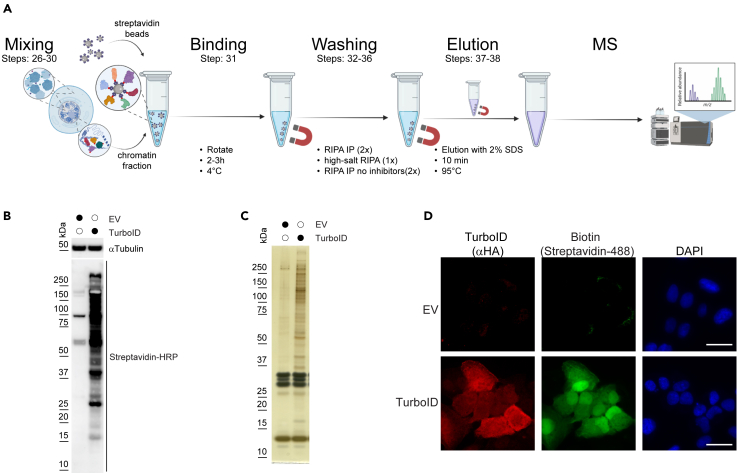
Overview of the affinity purification and quality control steps (A) Schematics showing the major steps of identifying DNA damage responders through affinity purification of biotinylated PCNA interactors. (B) Western blot showing whole-cell extracts of RPE1 cells transduced either with an empty vector (EV) or NLS-HA-TurboID. Lysates were probed with streptavidin-HRP to detect biotinylated proteins and with an anti-α-tubulin antibody as a loading control. (C) Silver staining of 5% of the eluates prepared from affinity purifications from EV and NLS-HA-TurboID transduced RPE1 cells. No high-salt wash was included during the purification process. (D) Immunofluorescence images of RPE1 cells transduced with either an EV or NLS-HA-TurboID. TurboID expression was detected using an anti-HA antibody, while biotinylated proteins were visualized with streptavidin-Alexa 488. Nuclear DNA was stained with DAPI. Scale bars represent 20 μm.

In this step, we perform an affinity purification to isolate biotinylated proteins that interact with PCNA, using streptavidin magnetic beads. Streptavidin binds biotin with exceptionally high affinity, enabling selective enrichment of proteins that were in close proximity to TurboID-PCNA during the labeling window. Steps 26–38 are summarized in Figure 1A.

***Note:*** To ensure reproducibility in downstream mass spectrometry analysis, prepare biological triplicates or quadruplicates. To minimize contamination from keratin and other sources, wear gloves, a face mask, safety goggles, and a head cover throughout the procedure. Use only pre-sterilized, protein-free tubes that have not been previously opened. Perform all steps in a clean bench to maintain sample integrity.

26. To equilibrate streptavidin beads, mix the MyOne C1 Streptavidin Magnetic Beads stock, to resuspend it fully before use.
27. Take 25 μL streptavidin beads slurry/ sample into a 1.5 mL microcentrifuge tube and add 1 mL of RIPA IP buffer.

***Note:*** Use 25 μL of streptavidin bead slurry per 15 cm plate per sample. For example, if you have 4 different samples and each was prepared from 4 × 15 cm plates (a total of 16 plates), you will need 400 μL of streptavidin bead slurry in total (16 plates × 25 μL).

28. Place the tube into a magnetic stand to collect the beads against the side of the tube. Remove supernatant.

***Note:*** Wait for 2 min before collecting the supernatant as these beads need time to completely get enriched at the side of the tubes. The DynaMag-2 magnetic stand enables complete buffer removal, as the magnets collect the beads on one side of the tube, allowing the buffer to be fully aspirated from the bottom of a 1.5 mL microcentrifuge tubes.

**CRITICAL:** Do not allow beads to dry at any step.

29. Resuspend the beads in 250 μL RIPA IP buffer / sample.

***Note:*** The 250 μL of RIPA IP buffer should contain streptavidin beads according to the number of plates used per sample, that is, 25 μL of bead slurry per 15 cm plate. For example, if a sample was derived from 4 plates, the 250 μL RIPA IP buffer will contain 100 μL resuspended bead slurry (4 × 25 μL).

30. To purify the biotinylated proteins, add the 250 μL equilibrated streptavidin beads to the 950 μL chromatin fraction prepared in Step 25.
31. Incubate the beads in the lysate at 4°C for 2–3 hours while gently rotating them.

***Note:*** To minimize foaming and mechanical stress on the lysate, use horizontal (gentle end-over-end) mixing instead of vertical agitation. Additionally, ensure that each 1.5 mL centrifuge tube contains at least 1 mL of liquid. This volume helps reduce protein precipitation caused by excessive foaming or shearing forces during mixing.

32. Wash the beads twice with 1 mL RIPA IP buffer.
  a. To perform this, place the tube into the magnetic stand and wait until the beads are collected on the side.
  b. Carefully remove the supernatant using a P1000 pipette without disturbing the beads.
  c. Add 1 mL RIPA IP buffer and repeat this step once more.
33. Wash the beads once with 1 mL high-salt RIPA IP buffer, following the same steps described above.

***Note:*** This high-salt washing step (with 500 mM NaCl) is necessary to remove Histone H1, that non-specifically associates with the magnetic beads (please check expected outcomes section).

34. Wash the beads once with 1 mL RIPA IP buffer without supplemented protease (Complete ULTRA Tablet) and phosphatase inhibitors (PhosSTOP).

**CRITICAL:** It is essential to remove protease inhibitors at this step to not have them in the final eluate. This allows the samples to be processed directly for MS without extra purification steps.

35. Transfer the beads to fresh 1.5 mL microcentrifuge tubes (optional, for reduced background).
36. Wash the beads once with 1 mL RIPA IP buffer without protease and phosphatase inhibitors.
37. To eluate the samples, resuspend the beads in 150 μL Elution buffer and boil them at 95°C for 10 minutes.
38. Place the samples onto a magnetic rack and collect the supernatant into a fresh tube. Discard the used beads. The eluate is now ready for direct MS analysis.

**Pause Point:** Eluates can be stored at −80°C for up to 6 months.

## Expected outcomes

The successful implementation of this protocol can be assessed at several points using different methods. A quick way to confirm whether cells express functional TurboID is to treat both parental RPE1 cells and TurboID-expressing stable RPE1 lines with biotin. Cell lysates can then be analyzed by Western blot using HRP-conjugated streptavidin to detect biotinylated proteins (Figure 1B). Alternatively, biotinylated proteins can be affinity-purified using streptavidin magnetic beads (as detailed in the protocol above), and the eluates visualized by silver staining. As shown in Figure 1C, TurboID-expressing cells display strong enrichment of biotinylated proteins compared to empty vector (EV) control. Figure 1C also shows prominent bands in both EV- and TurboID-expressing cells in the 25–30 kDa range. These bands can be selectively removed by including the high-salt RIPA IP buffer wash described in Step 33. Mass spectrometry analysis identified histone H1 as a major component of these bands (data not shown). Biotinylation can also be confirmed using immunofluorescence staining of cells, where biotinylated proteins are visualized with fluorophore-coupled streptavidin. The TurboID constructs described here also contain an HA epitope tag, enabling their detection with anti-HA antibodies (Figure 1D).

**Figure 2:**
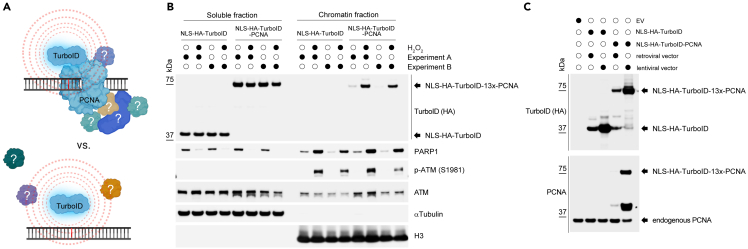
Characterization and validation of NLS-HA-TurboID-PCNA constructs and their chromatin recruitment under oxidative stress (A) Schematic illustrating the NLS-HA-TurboID-PCNA fusion construct used to biotinylate proteins in close proximity at sites of oxidative DNA damage (depicted as a red-marked base). (B) RPE1 cells stably expressing either NLS-HA-TurboID or NLS-HA-TurboID-PCNA were synchronized in G1 phase, then treated with biotin and H_2_O_2_ for 1.5 hours. Cells were subsequently fractionated into soluble and chromatin-bound protein fractions. Immunoblotting was performed to assess construct expression and chromatin recruitment. NLS-HA-TurboID-PCNA, but not the control NLS-HA-TurboID, is enriched in the chromatin fraction following oxidative stress, indicating damage-induced chromatin association. (C) Comparative Western blot showing NLS-HA-TurboID and NLS-HA-TurboID-PCNA expression levels using either retroviral or lentiviral vectors. A PCNA antibody was used to compare the expression of exogenous TurboID-tagged PCNA with endogenous PCNA levels. Asterix denotes non-specific band.

Figure 2A shows schematics of the TurboID constructs used to identify PCNA binders upon DNA damage. To verify *(i)* expression and *(ii)* DNA damage-induced chromatin enrichment of the transduced NLS-HA-TurboID and NLS-HA-TurboID-PCNA constructs, Western blotting was performed. The HA-tag enables comparison of NLS-HA-TurboID and NLS-HA-TurboID-PCNA expression levels. Figure 2B (soluble fraction) and 2C show comparable expression of both constructs, with lentiviral NLS-HA-TurboID-PCNA expressed at levels similar to endogenous PCNA.

As expected, only the NLS-HA-TurboID-PCNA construct is recruited to the chromatin fraction following oxidative damage, as detected by the anti-HA antibody (Figure 2B). Effective induction of oxidative DNA damage by H_2_O_2_ treatment can be confirmed by ATM activation, observed as increased phosphorylation at serine 1981 (pS1981-ATM) (Figure 2B). PARP1, which relocates to chromatin upon DNA damage, can serve as an additional control. Tubulin and histone H3 antibodies are used as loading controls and to assess the purity and efficiency of subcellular fractionation. Tubulin should only be observed in the soluble fraction while H3 should only be detectible in the chromatin fraction (Figure 2B).

The efficiency of cell cycle synchronization can be validated by both western blotting or FACS analysis of EdU incorporation combined with propidium iodide staining, as described in Rona et al,^1^ Figure S10.

**Figure 3:**
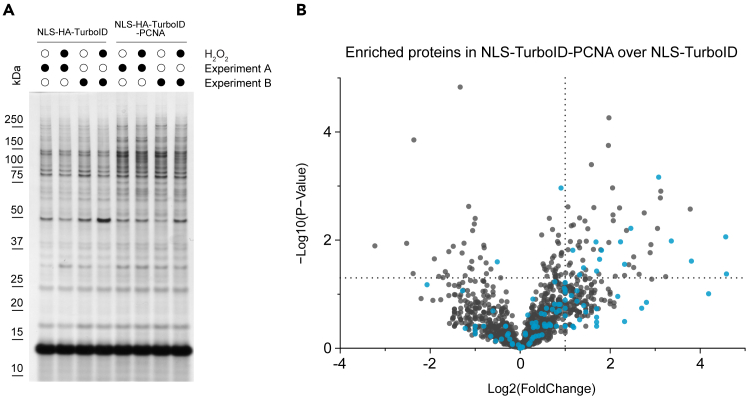
Binding partners of PCNA upon oxidative DNA damage in G1 (A) Silver stain of 5% of the streptavidin-purified eluates used for mass spectrometry reveals distinct biotinylated protein profiles in cells expressing NLS-HA-TurboID or the PCNA-fused construct. (B) Volcano plot comparing proteins enriched by NLS-HA-TurboID-PCNA relative to the NLS-HA-TurboID control. Established PCNA-binding partners, identified from the BioGRID database (access date: 7/12/22) are highlighted in blue and are significantly overrepresented among the enriched proteins (p = 2.42e-06, Fisher’s exact test). Hits were defined by a fold change ≥ 2 and an uncorrected p-value ≤ 0.05. MS spectra were searched against the human reference proteome from the UniProt database, supplemented with common laboratory contaminants and the sequences of the bait proteins. The complete list of quantified proteins, along with the PCNA-binding partner dataset used in the enrichment analysis, is provided in Rona et al.^1^ Supplementary Table S2. Raw mass spectrometry data are accessible via MassIVE (MSV000091006) and ProteomeXchange (PXD039228).

Finally, the affinity purified biotinylated protein fraction is illustrated in Figure 3. Although the silver staining pattern of NLS-HA-TurboID and NLS-HA-TurboID-PCNA samples appear only mildly different (Figure 3A), LC-MS reveals a clear and significant enrichment of PCNA interactors specifically in the TurboID-PCNA samples (Figure 3B). As expected, PCNA was also shown to increase its binding to several known DNA repair proteins upon H_2_O_2_ treatment, including BER and MMR factors.^1^ Please note that Figure 3A does not have strong non-specific bands around 25–30 kDa compared to Figure 1C, due to the use of the high-salt wash mentioned at Step 33.

## Limitations

Several potential limitations should be considered when using proximity biotinylation based approaches. TurboID has a molecular weight of ∼35 kDa, which may interfere with the function of the protein of interest due to its size. A smaller version, miniTurboID (∼28 kDa), is available and might cause less steric hindrance, although it is somewhat less enzymatically active than the full-length TurboID.^3^^,^^4^ Nevertheless, both TurboID and miniTurboID are significantly more efficient than earlier variants such as BioID or BioID2.^3^ New variants with varying sizes and activities are also being continuously developed.^5^

It is also important to evaluate which terminus, N- or C-terminal, is less likely to disrupt the native function of the protein when fused to TurboID. In addition to tag placement and size, the length and composition of the linker connecting TurboID to the protein of interest are crucial. TurboID has an effective labeling radius of approximately 10 nm, meaning longer linkers may capture more distant components of a complex,^3^^,^^6^ while shorter linkers may reduce background by keeping the biotinylation zone more localized. Flexible linkers, such as varying lengths of the (GGGGS)_n_ motif, are generally preferred.^7^^,^^8^

Using an untagged TurboID as a negative control during mass spectrometry helps minimize false-positive hits; however, validation using orthogonal methods is strongly recommended for any novel interactors. As with immunoprecipitation, detection by TurboID-based enrichment does not confirm a direct interaction between the protein of interest and its potential partner.

## Troubleshooting

### Problem 1

The tagged TurboID protein is not expressed, as it cannot be detected either via its epitope tag or through its biotinylation activity (related to Step 23 in the before you begin section).

### Potential solution

• Confirm successful viral transduction through antibiotic selection.
• Use a higher viral titer to improve the efficiency of generating stable cell lines.
• Consider using an alternative promoter to drive TurboID construct expression.
• If overexpression of your protein of interest is toxic, consider using an inducible expression system.

### Problem 2

The tagged TurboID protein is expressed, as confirmed by antibody staining, but does not show clear enrichment of biotinylated proteins based on streptavidin detection (related to Figure 1 and Step 38).

### Potential solution

• Tag the protein of interest at the opposite terminus to test whether it interferes less with TurboID function or accessibility.
• Use a longer, flexible linker to reduce potential steric hindrance between TurboID and the protein of interest.
• Allow TurboID labeling to proceed for at least 1 hour to ensure sufficient biotinylation.
• Optimize both the concentration and incubation time of biotin for efficient labeling.

### Problem 3

Excessive signal is observed in the empty vector (EV) control or in samples where no biotin was added (related to Step 23 in the before you begin section).

### Potential solution

• Incubate cells in biotin-free media for up to 96 hours to reduce background from endogenously biotinylated proteins.
• Use 100 μM biotin to induce TurboID-mediated biotinylation (Step 11). A concentration of 100 μM can be advantageous when cell confluency is higher than recommended in the protocol, as it helps prevent biotin depletion over time.

### Problem 4

Poor yield of biotinylated proteins in the biotin pull-down assay (related to Step 38).

### Potential solution

• Increase the amount of streptavidin beads used during affinity purification (Step 26–27).
• Start with a larger number of cells to boost protein yield.
• Ensure proper elution by boiling the beads in 2% SDS (Step 37); lower temperatures or lower SDS concentrations may be insufficient to disrupt the strong biotin-streptavidin interaction.

### Problem 5

Strong non-specific protein binding is observed on streptavidin beads around 25–30 kDa (related Figure 1C vs; Figure 3A).

### Potential solution

Use a high-salt wash (e.g., 500 mM NaCl) to remove non-specific binders (Step 33).

## Resource availability

### Lead contact

Further information and requests for resources and reagents should be directed to and will be fulfilled by the lead contact, Gergely Róna (rona.gergely@ttk.hu).

### Technical contact

Technical questions on executing this protocol should be directed to and will be answered by the technical contact, Roberta Borosta (borosta.roberta@ttk.hu).

### Materials availability

TurboID expression plasmids associated with this protocol are freely accessible through Addgene detailed in the before you begin section, Step 1.

### Data and code availability

• Proteomics data have been previously deposited at MassIVE (MSV000091006) and at the ProteomeXchange Consortium (PXD039228) and are publicly available.^1^
• Original data have been deposited to Mendeley Data: https://doi.org/10.17632/4gxswncytm.1. These data are publicly available as of the date of publication.
• Any additional information required to reanalyze the data reported in this paper is available from the lead contact upon request.

## Acknowledgments

We thank the members of the Rona Lab for helpful discussions. I.S.-N. is supported by the EKÖP-2024-124 New National Excellence Program and by the 2025-2.1.2-EKÖP-KDP-2025-00007 University Research Scholarship Programme of the Ministry for Culture and Innovation from the source of the National Research, Development and Innovation Fund. G.R. is supported by the Momentum Grant of the Hungarian Academy of Sciences (LP2023-15/2023), the EMBO Installation Grant (IG5670-2024), and the HUN-REN Welcome Home and Foreign Researcher Recruitment Grant (KSZF-143/2023).

## Author contributions

R.B. and G.R. conceptualized the study. R.B. and G.R. carried out the investigation and designed the protocol. I.S.-N. and I.K. provided figures and intellectual contribution. G.R. supervised, coordinated, and secured funding for the study. R.B. and G.R. wrote the paper with input from all authors.

## Declaration of interests

The authors declare no competing interests.

## Declaration of generative AI and AI-assisted technologies in the writing process

During the preparation of this work, the authors used GPT-4 to improve clarity and readability. All content generated with the assistance of this tool was thoroughly reviewed and edited by the authors, who take full responsibility for the final version of the manuscript.
